# Utilization of comprehensive health insurance scheme, Kerala: a comparative study of insured and uninsured BPL households

**DOI:** 10.1186/1753-6561-6-S5-P10

**Published:** 2012-09-28

**Authors:** Neena Elezebeth Philip, Kannan Srinivasan

**Affiliations:** 1 Sree Chitra Tirunal Institute for Medical Science and Technology, Trivandrum, India

## Introduction

Comprehensive Health Insurance Scheme (CHIS) in Kerala is a modification of the national social health insurance scheme called *Rashtriya Swasthya Bima Yojana* (RSBY). We aimed to (a) compare the socio-demographic and health utilization pattern (outpatient and inpatient services) of below poverty line (BPL) households insured under CHIS; (b) find the percentage hospitalization covered by CHIS; and (c) examine the out-of-pocket expenses for inpatient services.

## Methods

We conducted a cross-sectional survey of 149 insured and 147 un-insured BPL households, selected through systematic random sampling, in Kerala, India. Multivariate logistic regression, generalized estimating equation and Mann-Whitney U test were the statistical methods used.

## Results

Family size more than four (odds ratio (OR) 2.34, 95% confidence interval (CI) 1.13-4.82), chronic diseased family member (OR 2.05, 95% CI 1.18-3.57), high socio-economic status (OR 2.95, 95% CI 1.74-5.03) and an employed household head (OR 2.69, 95% CI1.44-5.02) were significantly associated with insured households. Both insured and uninsured households had similar utilization of outpatient services, but insured had higher hospitalisations (OR 1.57, 95% CI 1.05-2.34). Only 40% of the hospitalisation among the insured was covered by insurance. The mean out-of-pocket expenses for inpatient services among insured (INR 448.95) was significantly higher than that of the uninsured households (INR159.93), p = 0.003.

## Discussion

The major objective of CHIS, protecting poor people from financial catastrophe, was not achieved by the scheme. Even though CHIS has increased the utilization of the health care services, it did not enrol the poorest BPL households. These findings call for an urgent attention of the government to re-design and closely monitor the scheme.

## Competing interests

None declared

## Funding statement

The study was supported by the Health System Research Initiative India and OASIS.

